# MicroRNA expression profiling of peripheral blood mononuclear cells associated with syphilis

**DOI:** 10.1186/s12879-020-4846-x

**Published:** 2020-02-22

**Authors:** Tao Huang, Jun Zhang, Wujian Ke, Xiaohui Zhang, Wentao Chen, Jieyi Yang, Yiwen Liao, Fangwen Liang, Shuqing Mei, Mingjiu Li, Zhenzhou Luo, Qiwei Zhang, Bin Yang, Heping Zheng

**Affiliations:** 1 Dermatology Hospital, Southern Medical University, Guangzhou, China; 2 Cancer Research Institute, School of Basic Medical Sciences, Southern Medical University, Guangzhou, China; 3 Yingde Center for Chronic Disease Control, Yingde, China; 4 Zhuhai Center Chronic Disease Control, Zhuhai, China; 5 Panyu Institute of Chronic Disease, Guangzhou, China; 6 Shenzhen Nanshan Center for Chronic Disease Control, Shenzhen, China; 7 Department of Microbiology, School of Public Health, Southern Medical University, Guangzhou, China

**Keywords:** Syphilis, microRNA profiling, Peripheral blood mononuclear cells, *Treponema pallidum*, Sexually transmitted infections

## Abstract

**Background:**

*Treponema pallidum* (*T. pallidum*) infection evokes significant immune responses, resulting in tissue damage. The immune mechanism underlying *T. pallidum* infection is still unclear, although microRNAs (miRNAs) have been shown to influence immune cell function and, consequently, the generation of antibody responses during other microbe infections. However, these mechanisms are unknown for *T. pallidum*.

**Methods:**

In this study, we performed a comprehensive analysis of differentially expressed miRNAs in healthy individuals, untreated patients with syphilis, patients in the serofast state, and serologically cured patients. miRNAs were profiled from the peripheral blood of patients obtained at the time of serological diagnosis. Then, both the target sequence analysis of these different miRNAs and pathway analysis were performed to identify important immune and cell signaling pathways. Quantitative reverse transcription-polymerase chain reaction (RT-PCR) was performed for microRNA analysis.

**Results:**

A total of 74 differentially regulated miRNAs were identified. Following RT-qPCR confirmation, three miRNAs (hsa-miR-195-5p, hsa-miR-223-3p, hsa-miR-589-3p) showed significant differences in the serofast and serologically cured states (*P* < 0.05). One miRNA (hsa-miR-195-5p) showed significant differences between untreated patients and healthy individuals.

**Conclusions:**

This is the first study of miRNA expression differences in peripheral blood mononuclear cells (PBMCs) in different stages of *T. pallium* infection. Our study suggests that the combination of three miRNAs has great potential to serve as a non-invasive biomarker of *T. pallium* infections, which will facilitate better diagnosis and treatment of *T. pallium* infections.

## Background

Syphilis is caused by infection with the spirochete *Treponema pallidum* subsp. pallidum (*T. pallidum*) [1, 2]. It is one of the most common sexually transmitted diseases worldwide. Syphilis is a multistage progressive disease with a variety of manifestations, including chancre, disseminated skin lesions, gummas, neurosyphilis, and cardiovascular syphilis [2]. Syphilis symptoms usually resolve with appropriate antibiotic medications. However, the evaluation of a therapeutic response requires serological testing. Patients with nontreponemal titers that decline 4-fold or more are considered as having a good serological response, whereas those with neither an increase nor 4-fold decrease are referred to as being “serofast” [3]. The proportion of serofast patients has reached 15–41% [4]. It is unknown why symptoms and severity vary so greatly among syphilis patients or why serological reactions are not mitigated in serofast patients, but they are likely the outcome of host immune responses elicited by *T. pallidum*. Although a new study showed successful culture of *T. pallidum* in vitro [5], it is still difficult to culture in vitro. Thus, the pathogenesis of syphilis is not yet clear. The current diagnostic methods for syphilis cannot distinguish between the serofast state and latent syphilis. Many researchers are currently attempting to develop new biomarkers for diagnosis.

Macrophages have been shown to be activated during syphilis infection, as demonstrated by the production of macrophage-activating factors (MAFs) from syphilitic rabbits [6]. Dendritic cells (DCs), which are the most potent antigen-presenting cells, can phagocytize *T. pallidum* and produce inflammatory cytokines, including interleukin 1β (IL-1β), IL-6, and tumor necrosis factor alpha (TNF-α) [7], which are crucial for the initiation of T cell responses to *T. pallidum* infection. Evidence of T lymphocyte infiltration of syphilitic lesions was provided by Engelkens et al. [8]. Previous studies have demonstrated that the Th1 cytokines IL-2, IL-12, and gamma interferon (IFN-γ) were predominantly expressed by both the infiltrating T cells in lesions [9] and splenic lymphocytes stimulated by sonicated *T. pallidum* [10]. However, the Th1 response is suppressed by Th2 cytokine IL-10 with the progression to latent syphilis [11], which is characteristic of a strong Th2-mediated humoral immune response. Immunosuppression also occurs in syphilitic serofast patients with evidence of obviously increasing numbers of regulatory T cells (Treg), which have potent immunosuppressive activity [12]. However, the mechanism underlying immune regulation in syphilis infection remains unclear. The abnormalities of immune cells in syphilis were induced in a complex manner involving genomic and transcriptomic changes. Many studies have established that pathogens can affect host immunity by regulating host microRNA expression.

MicroRNAs (miRNAs) are evolutionarily conserved small noncoding RNA molecules. The sequence of microRNAs usually includes 19–24 nucleotides. They can bind to the target mRNA, resulting in translational suppression or degradation of mRNA [13]. Due to the important function of microRNAs, they regulate approximately 30% of the gene transcription involved in a variety of cellular processes, including the immune response to invading pathogens [14]. The miRNAs (i.e., miR-223-3p, miR-150, miR-146b, miR-16, and miR-191), abundantly expressed in T cells, were down-regulated in human immunodeficiency virus (HIV) patients [15]. Moreover, HIV-1 gp120 could activate the STAT3 signaling pathway to regulate the expression of miRNA-21, miRNA-155, and miRNA-181b in monocyte-derived dendritic cells (MDDCs) [16]. Six serum miRNAs (miRNA-378, miRNA-483-5p, miRNA-22, miRNA-29c, miRNA-101, and miRNA-320b) have been reported to be differentially expressed in tuberculosis, which is associated with the regulation of some target genes associated with mitogen-activated protein kinases (MAPK) and TGF-β signaling [17]. Altered expression of miRNA-155, related to immune activation and inflammation, was found to be regulated by IL-10, thus playing an important role in the suppression of *Borrelia burgdorferi*-induced Lyme arthritis and carditis [18]. These studies highlight the importance of altered miRNAs in the immune response associated with infectious diseases. However, the study of miRNA profiles in syphilis is still limited.

This is the first study of miRNA expression differences in peripheral blood mononuclear cells (PBMCs) in different stages of *T. pallium* infection. In our recent study, miRNA levels in serum increased after *T. pallium* infection, which verified that miRNA-19b-3p was downregulated and related to the suppression of Th1 production in syphilis [19]. In the present study, we aimed to use microarray analysis to detect differential miRNA expression in PBMCs from syphilis patients to attempt to identify the key molecule in dysfunctional immune cells. Several miRNAs have the potential to become novel biomarkers for syphilis diagnosis and prognosis.

## Methods

### Sample quality control and principal component analysis

Peripheral blood samples were obtained from individuals who visited the Dermatology Hospital of Southern Medical University, Guangzhou Panyu Center for Chronic Disease Control, Zhuhai Center for Chronic Disease Control, Yingde Center for Chronic Disease Control, and Shenzhen Nanshan Center for Chronic Disease Control. All samples were diagnosed using the rapid plasma reagin test (RPR) and *T. pallidum* particle agglutination assay (TPPA). According to Chinese syphilis prevention and treatment guidelines (version 2015), patients in the serofast state were defined as infected patients with a positive TPPA and a low-positive RPR result for 1 year after standard treatment. Serological cure was defined as a positive TPPA and negative RPR result.

The exclusion criteria were as follows: 1) patients who were co-infected with HIV, condyloma acuminata, or other sexually transmitted diseases; and 2) patients who were suffering from autoimmune disease, were undergoing anti-inflammatory or immunosuppressive therapy, or had taken antibiotics within the past 6 months.

This study was approved by the Ethics Committee at the Guangdong Provincial Dermatology Hospital. The objectives, procedures, and potential risks were verbally explained to all participants. Written informed consent was obtained from all patients prior to inclusion in this study.

### RNA extraction

PBMCs were isolated from whole blood using a standard procedure of Ficoll gradient centrifugation, performed in strict accordance with the manufacturer’s instructions. Total RNA in PBMCs was extracted using Trizol reagent. RNA was quantified using a NanoDrop One spectrophotometer (Thermo Fisher Scientific, Waltham, MA, USA). RNA was suspended in RNase-free water and stored at − 80 °C.

### Microarray analysis

Fluorescent targets were prepared from 2.5 μg total RNA samples using miRNA ULSTM Labeling Kit (Kreatech Diagnostics, The Netherlands). Labeled miRNA targets enriched by NanoSep 100 K (Pall Corporation, USA) were hybridized to the Human miRNA OneArray® v5.1 follow the manufacturer’s instructions. After 16 h hybridization at 37 °C, non-specific binding targets were washed away by three different washing steps (WashI 37 °C 5 mins; Wash II37 °C, 5 mins 25 °C 5 mins; Wash III rinse 20 times), and the slides were dried by centrifugation and scanned by an Axon 4000B scanner (Molecular Devices, Sunnyvale, CA, USA). The Cy5 fluorescent intensities of each spot were analyzed by GenePix 4.1 software (Molecular Devices).

The signal intensity of each spot was process by R program. We filtered out spots that the flag < 0. Spots that passed the criteria were normalized by 75% media scaling normalization method. Normalized spot intensities were transformed to gene expression log2 ratios between the control and treatment groups. The genes with |log2 ratio| ≥ 0.585 (FC ≥ 1.5) and *P*-value < 0.05 are selected to further studies.

### MicroRNA-predicted target genes, gene ontology (GO), and pathway analyses (ref: clinical epigenetics, 2017, 9, 79)

Potential target miRNAs were predicted and analyzed using bioinformatics algorithms (miRWalk, DIANA-microT4, miRanda, miRDB, PICTAR2, and TargetScan) with miRWalk2.0 [20]. To reduce the number of false-positives, only target genes that were predicted by at least four of the six programs were selected and used for further investigation. The biological annotation and the potential pathways were analyzed using DAVID, version 6.7, and KEGG pathway enrichment analysis, respectively.

### Quantitative RT-PCR

We evaluated 12 healthy controls and 94 syphilis patients using RT-PCR to verify the changes in miRNA expression using quantitative RT-PCR. A total of 49 current syphilis patients had not been treated with antibiotics before enrollment, including patients with primary (*n* = 14), secondary (*n* = 18), early latent (*n* = 10), and late latent syphilis (*n* = 7) infections. In addition, 45 patients were divided into two groups following standard treatment: serofast state (*n* = 28) and serologically cured (*n* = 17). All clinical information is provided in Table 2.

In order to simulate the Tp infection of PBMCs in the experiment in vitro, Tp is incubated with PBMCs of healthy individuals in culture plates. We collected PBMCs from 8 healthy individuals in this experiment.

Quantitative RT-PCR (RT-qPCR) analysis was performed using a Mir-X miRNA RT-qPCR SYBR Kit (Takara Biomedicals, Shiga, Japan) according to the manufacturer’s instructions. Briefly, each 1 μg of total RNA was reverse-transcribed to cDNA using the Mir-X miRNA First-Strand Synthesis Kit (Takara Biomedicals). MicroRNA expression was analyzed in duplicate and normalized to U6 on a LightCycler 480 (Roche Applied Science, Basel, Switzerland). The fold change for microRNA was calculated using the comparative-Ct (ΔCt) method.

### Statistics

Statistical analyses were performed using GraphPad Prism software 5.01. Data are presented as the mean ± standard deviation (SD) unless otherwise stated. Student’s t-test was used for two-group comparisons. Significance of the microarray analysis between multiple groups was determined by one-way analysis of variance (ANOVA) with Tukey’s multiple comparison test. A *P* value < 0.05 was considered statistically significant.

## Results

### Sample quality control and principal component analysis

**Fig. 1:**
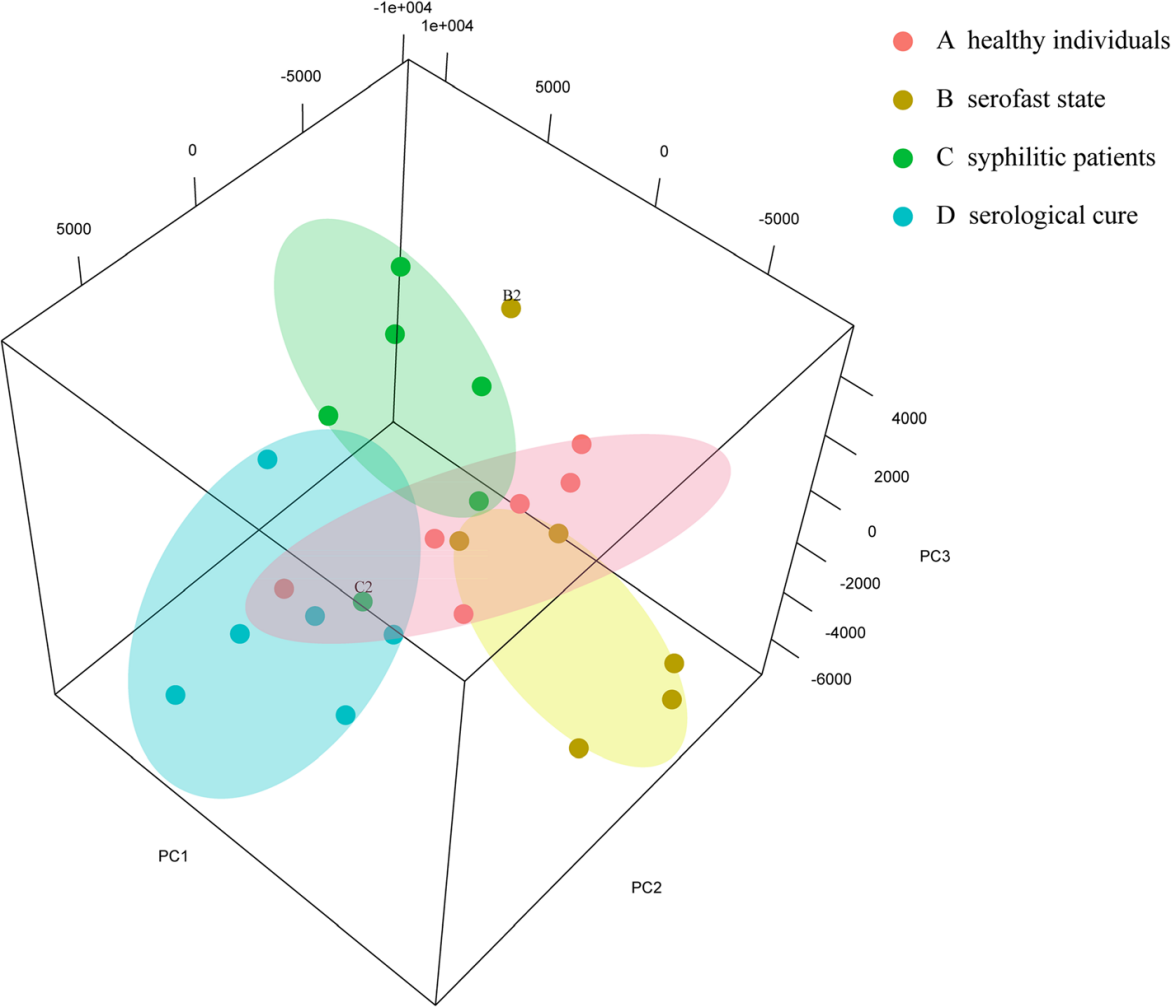
Principal component analysis (PCA) of miRNA expression data from human peripheral blood mononuclear cells (PBMCs). Red, A1-A6, healthy individuals; yellow, B1-B6, serofast state patients; green, C1-C6, syphilis patients before treatment; blue, D1-D6, serologically cured patients

As a means of quality control to assess the biological separation of the groups based on probe intensities, principal component analysis (PCA) was performed using Partek Genomics software. Four sample groups were clearly observed, namely, heathy volunteers, syphilis patients before treatment, serofast-state syphilis patients, and serologically cured syphilis patients (Fig. 1). The principal component of the serologically cured group was significantly different from that of the serofast-state group. The principal components of the groups were different. B2 was very different from that of the group of serofast state patients. C3 was also different from that of the group of syphilis patients before treatment.

### Differentially expressed MiRNAs

**Fig. 2:**
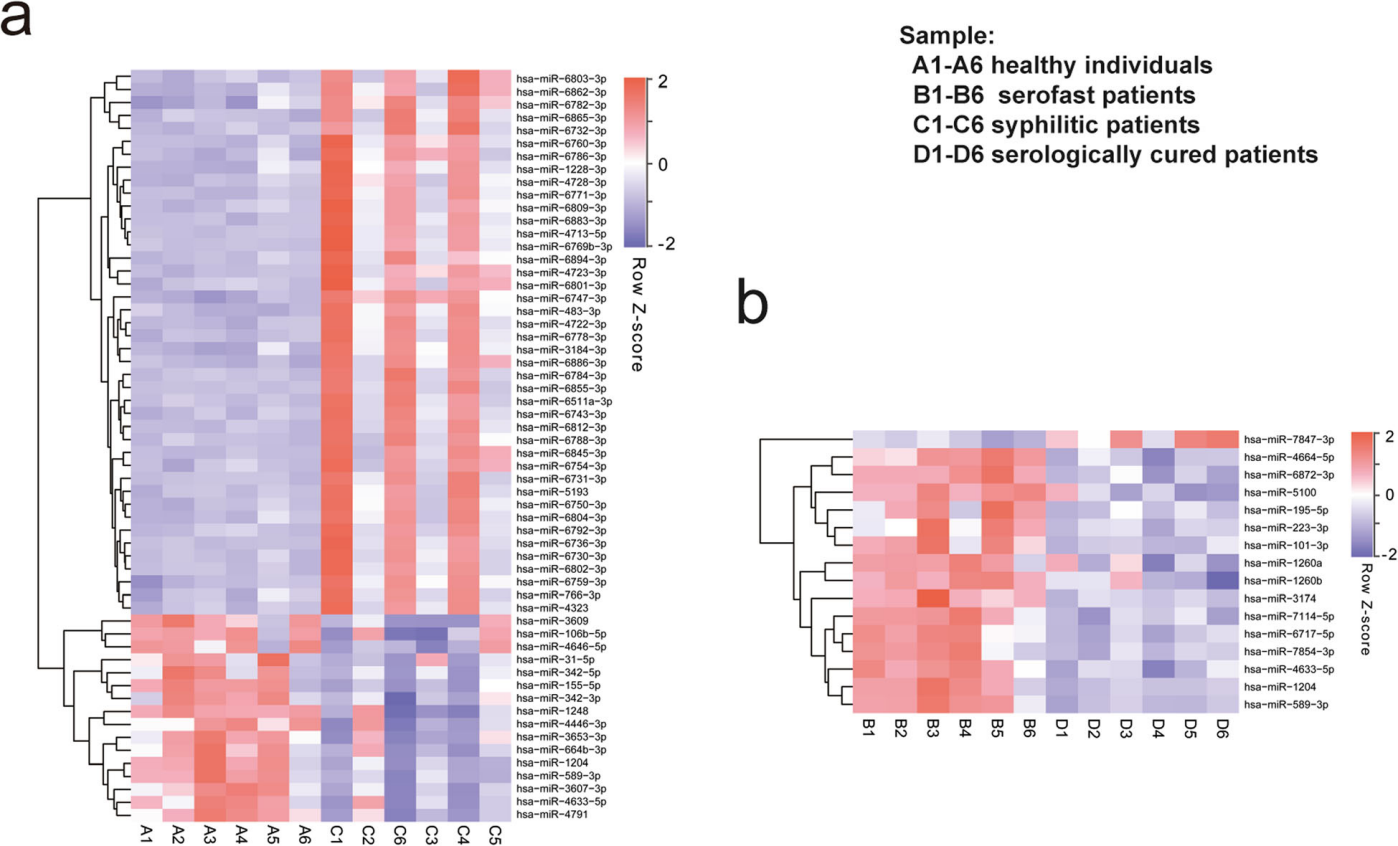
miRNA screening in healthy individuals(A1-A6), syphilitic patients (C1-C6), serofast patients (B1-B6), and serologically cured patients (D1-D6). Red boxes indicate up-regulated miRNAs, and purple boxes indicate down-regulated miRNAs. The brightness indicates the magnitude of the difference. Changes in miRNA expression (FC > =1.5, *P* < 0.05) are illustrated by the heat map. For interpretation of the colors in this figure legend, the reader is referred to the web version of this article. **a** Comparison of syphilitic patients and healthy individuals, **b** Comparison of serofast patients and serologically cured patients

We performed differential miRNA assays of the PBMCs from six healthy individuals, six untreated syphilitic patients (three with primary syphilis and three with secondary syphilis), six serofast patients, and six serologically cured patients. The clinical information for all specimens is summarized in Table 1. We compare the differential expression of before treatment (syphilis patients and healthy individuals) and after treatment (serofast state and serofast patients compared with the serologically cured patients). Compared with the results from healthy individuals, 42 miRNAs were up-regulated and 16 miRNAs were down-regulated in the untreated patients with syphilis (Fig. 2a). Meanwhile, 15 miRNAs were down-regulated and one miRNA was up-regulated in the serologically cured patients compared with the serofast patients (Fig. 2b).

**Table 1:**
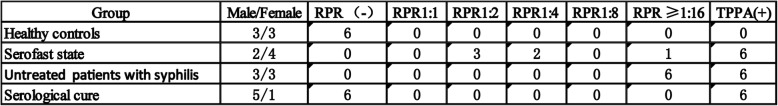
Information on the clinical samples used for the array experiments.

### Differential gene target sequence prediction and pathway enrichment analysis

**Fig. 3:**
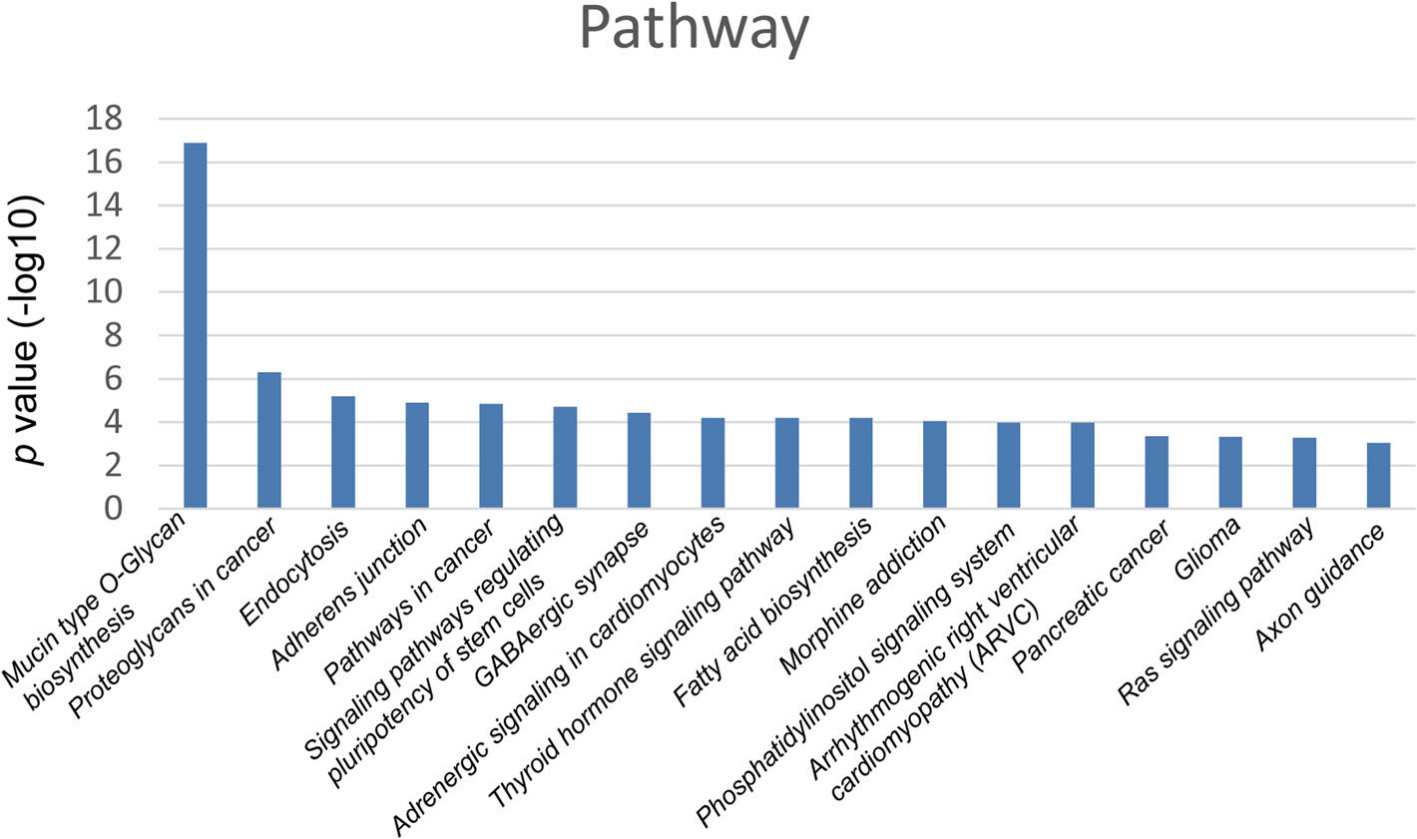
Pathway analysis. The top 17 most significantly changed pathways associated with target genes. The Y-axis shows the negative logarithm of the *P* value (−lg p), and the blue bars show the changed pathways

To understand the possible functions of these differential miRNAs, the target sequences of differentiated miRNAs among the groups were predicted using the Targetcan database. The target genes were then subjected to KEGG pathway enrichment analysis. As shown in Fig. 3, there are several possible regulatory pathways of 17 discrepant miRNAs that meet the minimum *P* value. These pathways were mucin type O-Glycan biosynthesis, proteoglycans in cancer, endocytosis, adherens junctions, pathways in cancer, signaling pathways regulating pluripotency of stem cells, GABAergic synapses, fatty acid biosynthesis, the thyroid hormone signaling pathway, adrenergic signaling in cardiomyocytes, morphine addiction, the phosphatidylinositol signaling system, arrhythmogenic right ventricular cardiomyopathy (ARVC), pancreatic cancer, glioma, the Ras signaling pathway, and axon guidance pathways.

### Bioinformatics network analysis of candidate miRNAs and corresponding target sequences

**Fig. 4:**
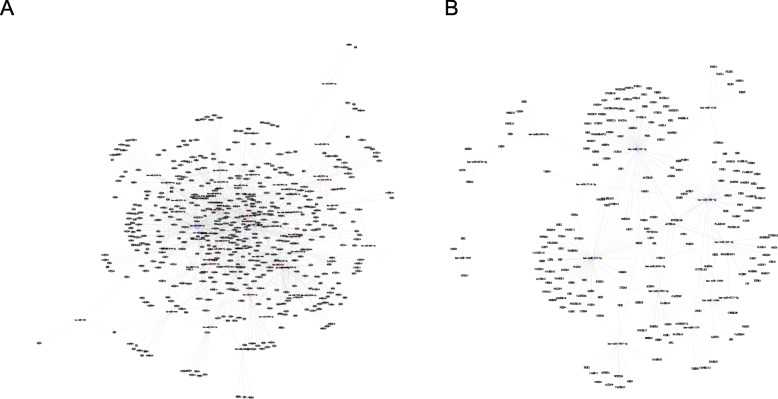
MicroRNA-gene network. The microRNA-gene network demonstrated that the predicted target genes were regulated by miRNAs. Square grid nodes represent microRNAs, cycle nodes represent target genes, red indicates upregulated genes, and blue indicates downregulated genes. The size of the circle or square represents the degree value. Larger circles are associated with miRNAs that play more critical roles in regulation. **a** Red represents syphilitic patients up-regulated relative to healthy individuals, blue opposite. **b** Red represents serologically cured patients up-regulated relative to serofast state, blue opposite

To understand the role of miRNAs and corresponding target genes in syphilis infection, we conducted an interplay analysis between miRNAs and corresponding target genes. The results could help to better explain the key regulatory functions of miRNAs. Figure 3 shows 17 pathways with the lowest *P* values (most relevant) of discrepant miRNAs and corresponding target genes. Among them, the miRNAs of 497 target genes showed differences between syphilitic patients and healthy individuals (Fig. 4a). Additionally, 213 target genes showed differences in miRNA expression between serologically cured patients and serofast patients. There were 15 down-regulated miRNAs and one up-regulated miRNA (Fig. 4b). Details of target genes are presented in Additional file 1: Table S1.

### The expression of seven miRNAs in clinical specimens

**Fig. 5:**
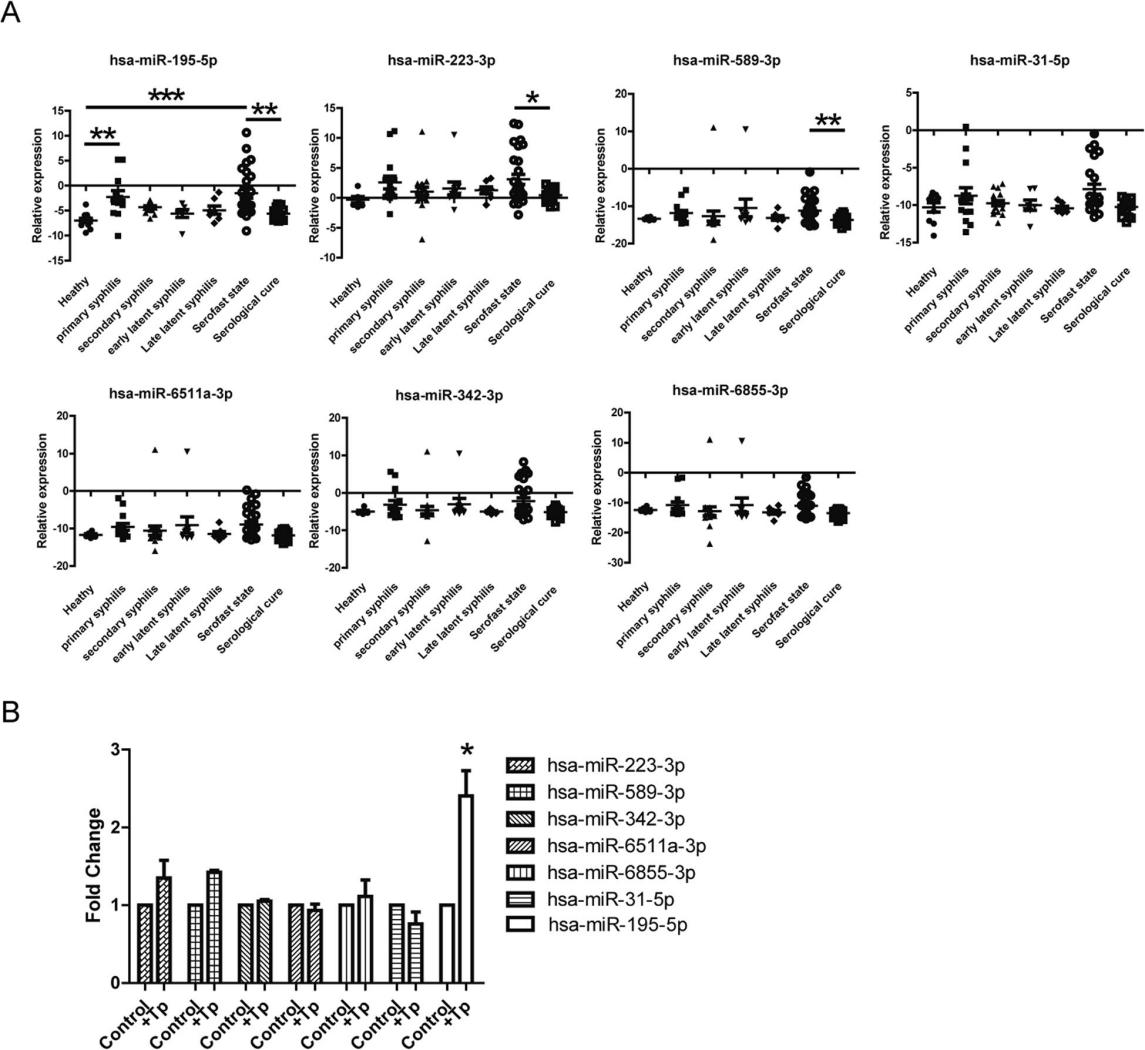
**a** Detection of PBMCs of different stages miRNAs by the RT-qPCR assay. The expression of eight miRNAs was measured in 106 samples. We analyzed the expression of eight miRNAs (hsa-miR-195-5p, hsa-miR-223-3p, hsa-miR-589-3p, hsa-miR-342-3p, hsa-miR-6511a-3p, hsa-miR-31-5p, hsa-miR-6855-3p) selected from the microarray data by using RT-PCR. Relative expression was used to normalize the relative gene expression data in the RT-qPCR assay. U6 was set as the reference gene. Statistical analysis was performed using the nonparametric Mann-Whitney test. ***P < 0.001, **P < 0.01, *P < 0.05. **b** PBMCs from healthy individuals were incubated with *T. pallidum*, and the expression of eight miRNAs was measured by RT-PCR

To verify the relationship between these miRNAs and syphilis, 106 clinical specimens were used to assess the differential expression of miRNAs. Seven differentially expressed miRNAs were selected. These miRNAs were selected from the comparison of healthy and syphilitic patients, serofast state and serological cure, respectively. The expression of miR-6511a-3p, miR-6855-3p, miR-31-5p, miR-342-3p and miR-589-3p were significant different in healthy persons and syphilitic patients. And the expression of miR-195-5p, miR-223-3p and miR-589-3p were significant different in serofast state and serological cure. The clinical information for all specimens is summarized in Table 2. Figure 5 shows the expression of seven miRNAs in each group. The expression of miR-195-5p in the serofast patients was significantly higher than that in the serologically cured and healthy individuals. It was also higher in primary syphilis patients than in healthy individuals. There was no significant difference between the other groups. In addition, the expression of miR-223-3p and miR-589-3p in the serofast patients was significantly higher than that in the serologically cured patients (Fig. 5a). These data indicate that there is no significant difference between early latent syphilis and late latent syphilis among most miRNAs. Additionally, there was no significant difference in the expression of other miRNAs. Furthermore, PBMCs of healthy individuals were incubated with *T. pallidum*, and the data showed that miR-195-5p was up-regulated following *T. pallidum* incubation (Fig. 5b).

**Table 2:**
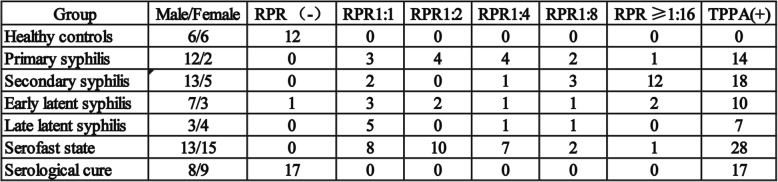
Information on the clinical samples used in the qPCR experiments.

These results suggested that miR-195-5p, miR-589-3p, and miR-223-3p may be related to the pathogenesis of the serofast state. Meanwhile, miR-195-5p was related to *T. pallidum* infection.

## Discussion

*T. pallidum* remains one of the human pathogens that it is still difficult to culture in vitro. There is only a defective animal research model for it. These obstacles have greatly hindered the effort to elucidate the basic immunobiological traits of syphilis. *T. pallidum*-specific antibodies are not significantly altered after treatment and have no detectable protective effect. Patients in the serofast state and patients with latent syphilis exhibit a similar serological response. Serological diagnosis cannot distinguish the serofast state from latent syphilis, and the serofast state is not uncommon clinically. There is no evidence that the serofast state is relevant to *T. pallidum*. Therefore, how to treat and identify the serofast state are very important issues associated with the prevention of syphilis. At the same time, the immune mechanism of syphilis is not clear. Several studies have shown that immunosuppression can occur after infection with *T. pallidum*. However, the mechanism of immune regulation is unclear.

Recently, miRNAs have been intensively studied as new biomarkers for diagnosis and prognosis in various diseases, such as cancers, heart disease, diabetes, psychosis, and infectious diseases [20–24]. Many researchers studying the expression profile of infectious diseases have shown that microRNAs play an important role in the host’s anti-infective immune response. This study provides the first miRNA expression profile of peripheral blood samples from healthy individuals, untreated syphilis patients, patients in the serofast state, and serologically cured patients. In a recent study [19], we used microarray analysis to assess the differential serum miRNA expression profile in syphilis patients and matched healthy controls. Among the differentially expressed microRNAs identified by microarray analysis, miR-21-5p, miR-19b-3p, miR-16-5p, and miR-142-3p were selected as candidates for further testing using RTq-PCR. miRNAs in serum may be derived from PBMCs. So we compared the profiles of miRNAs PBMCs and serum. Unfortunately, no consistent miRNAs were found. Therefore, we did not measure those miRNAs that are differentially expressed in serum in this project.

In this study, we found 74 differentially expressed miRNAs. According to the microarray analysis, 42 miRNAs in untreated syphilis patients were up-regulated relative to those in healthy individuals, and 16 miRNAs were down-regulated. One miRNA in the serologically cured patients were up-regulated relative to the serofast patients, and 15 miRNAs was down-regulated. However, we did not find the same differential miRNA expression in the serum analysis. Many miRNAs were expressed in untreated syphilis patients compared with healthy controls, indicating that *T. pallidum* infection can lead to changes in immune mechanisms.

miRNAs regulate gene expression by binding to complementary sites on mRNAs and reducing mRNA stability and translation [25]. miRNAs could regulate gene expression and serve as transcription factors by regulating the development timing and differentiation of cells. Alteration in miRNA expression may have affected the signaling pathway. Pathway analysis aided our assessment of the biological processes involved in immune responses of miRNAs and target genes. In this study, pathway analysis indicated that predicted target genes for those miRNAs were involved in mucin type O-glycan biosynthesis, proteoglycans in cancer, endocytosis, adherens junctions, pathways in cancer, signaling pathways regulating pluripotency of stem cells, GABAergic synapses, fatty acid biosynthesis, the thyroid hormone signaling pathway, adrenergic signaling in cardiomyocytes, morphine addiction, the phosphatidylinositol signaling system, arrhythmogenic right ventricular cardiomyopathy (ARVC), pancreatic cancer, glioma, the Ras signaling pathway, and axon guidance pathways. Adherence-mediated colonization plays an important role in the pathogenesis of microbial infections, particularly those caused by extracellular pathogens responsible for systemic diseases, such as *T. pallidum* subsp. pallidum, the agent of syphilis. Many studies have shown that outer membrane proteins of *T. pallidum*, such as TP0136, TP0155, and TP0483, could participate in the adhesion mechanism [26, 27]. Natural immunity is the first barrier after *T. pallidum* infection. A variety of lipoproteins of *T. pallidum* activate phagocytes and dendritic cells (DCs) through the CD14, toll-like receptor 1 (TLR1) and TLR2-dependent signaling pathways, and these pathogen-associated pattern molecules (PAMPs) are thought to be the main pro-inflammatory factors in the process of *T. pallidum* infection. Special outer membrane structures of *T. pallidum* that lack exposed surface lipoproteins cause PAMPs to be refractory to TLRs or other pattern recognition receptors (PRRs) of macrophages or DCs. In this manner, innate immunity cannot be activated, and *T. pallidum* cannot be cleared by the immune system [28]. The miRNAs of these signaling pathways may be suitable targets for research regarding syphilis immunomodulation.

The miRNA-gene network helped us to screen miRNAs important in regulating immune response. This study demonstrated that miRNAs may play an important role in the regulation of syphilis-related immune mechanisms, such as miR-195-5p and miR-223-3p. We used RTq-PCR to verify the expression of different miRNAs, and a significant difference was found in the expression of three miRNAs (hsa-miR-195-5p, hsa-miR-223-3p, and hsa-miR-589-3p) of PMBCs in 106 samples. First, we were interested in miR-195-5p. Most studies on miR-195-5p focus on the regulatory role of apoptosis, which can inhibit the expression of some anti-apoptotic proteins [29–32]. One recent study showed that miR-195-5p can inhibit the proinflammatory expression of macrophages [33]. Interestingly, miR-195-5p was found to be differentially expressed between serofast state patients and latent syphilis patients (all with early latent and late latent syphilis, data not shown). Furthermore, miR-195-5p was up-regulated after *T. pallidum* incubation in PBMCs from healthy individuals. These data suggest that miRNAs are associated with *T. pallidum* infection. In addition, miRNA-223-3p can inhibit the proinflammatory responses in *Helicobacter pylori* infection-related macrophages [34]. Macrophage-mediated inflammation can activate cellular immunity. It appears to affect the syphilis-related immune mechanism by affecting the apoptosis of immune cells, and it is involved in the regulation of syphilis-related inflammation.

The causes of the serofast state are the subject of some debate. Some studies posit that this state is due to the incomplete elimination of *T. pallidum*. However, there is no evidence that *T. pallidum* is latent. RT-qPCR data indicate that individual differences in serological cure are small. However, individual differences in the serofast state are more pronounced. We think this discrepancy is due to the unclear definition of the serofast state. The results of serological testing of patients in the serofast state are very similar to those of patients with latent syphilis. There are also many complicated questions regarding the serofast state that need to be addressed by syphilis researchers. Studying the regulation of miRNAs in immunity can improve our understanding of the serofast state.

## Conclusions

In summary, our results suggest that changes in miRNA expression profiles may be associated with immune tolerance and persistent *T. pallidum* infection through regulation of target genes or signaling pathways. miRNAs that are differentially expressed among syphilis patients, such as miRNA-195-5p, might be new biomarkers for *T. pallidum* persistence, and they may be good candidates for investigation of the mechanism of *T. pallidum* clearance. Blocked or decreased expression of these miRNAs may have some role in the treatment of syphilis and management of the serofast state.

## Supplementary information

**Additional file 1.** Information on target genes of differential miRNAs.

## Data Availability

The datasets generated and/or analyzed during the current study are available in NCBI’s Gene Expression Omnibus https://www.ncbi.nlm.nih.gov/geo/ and are accessible through GEO Series accession number GSE142676.

## Abbreviations

IL-1β/IL-6: Interleukin 1β/6
miRNA: MicroRNA
PBMCs: Peripheral blood mononuclear cells
PCA: Principal component analysis
RPR: Toluidine red unheated serum regain test
RT-PCR: Quantitative reverse transcription-polymerase chain reaction
TLR1/2: Toll-like receptor 1/2
TNF-α: Tumor necrosis factor alpha
*Tp/ T. pallidum*: *Treponema pallidum*
TPPA: *T. pallidum* particle agglutination assay
